# “Provide care for everyone please”: engaging community leaders as sexual and reproductive health advocates in North and South Kivu, Democratic Republic of the Congo

**DOI:** 10.1186/s12978-019-0764-z

**Published:** 2019-07-08

**Authors:** Victoria J. Steven, Julianne Deitch, Erin Files Dumas, Meghan C. Gallagher, Jimmy Nzau, Augustin Paluku, Sara E. Casey

**Affiliations:** 1 RAISE Initiative, Heilbrunn Department of Population and Family Health, Mailman School of Public Health, Columbia University, 60 Haven Ave, B2, New York, NY 10032 USA; 2 CARE USA, 151 Ellis St NE, Atlanta, GA 30303 USA; 3 Save the Children, 899 North Capitol Street, Suite 900, Washington, DC 20002 USA; 4 International Rescue Committee, 63, Ave Colonel Mondjiba, Kinshasa, Democratic Republic of the Congo

**Keywords:** Abortion, Contraception, Community leader, Post-abortion care, DRC, Qualitative, Community mobilization

## Abstract

**Background:**

Inadequate infrastructure, security threats from ongoing armed conflict, and conservative socio-cultural and gender norms that favour large families and patriarchal power structures contribute to poor sexual and reproductive health (SRH) outcomes in North and South Kivu provinces, Democratic Republic of the Congo (DRC). In order to expand contraceptive and post-abortion care (PAC) access in North and South Kivu, CARE, the International Rescue Committee and Save the Children provided technical support to the Ministry of Health and health facilities in these regions. Partners acknowledged that community leaders, given their power to influence local customs, could play a critical role as agents of change in addressing inequitable gender norms, stigma surrounding SRH service utilization, and topics traditionally considered taboo within Congolese society. As such, partners actively engaged with community leaders through a variety of activities such as community mapping exercises, values clarification and transformation (VCAT) activities, situational analyses, and education.

**Methods:**

This manuscript presents findings from 12 key informant interviews (KIIs) with male political and non-political community leaders conducted in six rural health zones of North and South Kivu, DRC. Transcripts were analysed thematically to explore community leaders’ perceptions of their role in addressing the issue of unintended pregnancy in their communities.

**Results:**

While community leaders in this study expressed overall positive impressions of contraception and strong support for ensuring access to PAC services following spontaneous and induced abortions, the vast majority held negative beliefs concerning women who had induced abortion. Contrasting with their professed opposition to induced abortion, leaders’ commitment to mediating interpersonal conflict arising between community members and women who had abortions was overwhelming.

**Conclusion:**

Results from this study suggest that when thoughtfully engaged by health interventions, community leaders can be empowered to become advocates for SRH. While study participants were strong supporters of contraception and PAC, they expressed negative perceptions of induced abortion. Given the hypothesized link between the presence of induced abortion stigma and care-avoidance behavior, further engagement and values clarification exercises with leaders must be integrated into community mobilization and engagement activities in order to increase PAC utilization.

## Plain English summary

Access to comprehensive sexual and reproductive health (SRH) services, including modern contraceptive methods and post-abortion care (PAC), can greatly reduce maternal mortality and morbidity. CARE, the International Rescue Committee, and Save the Children provided technical support to the Ministry of Health in North and South Kivu, Democratic Republic of the Congo (DRC) to improve contraceptive and PAC services. In addition to delivering training, supervision, and supplies, these three organizations have also invested significant resources into community engagement to address normative barriers to women’s access to SRH care and increase demand for SRH services. As some SRH issues are often seen as taboo in these communities, partners prioritized collaborating with local leaders to address socio-cultural barriers to utilizing SRH services and lend credibility to the partners’ SRH activities.

This paper presents findings from 12 key informant interviews (KIIs) with community leaders conducted in North and South Kivu, DRC. Results from this study suggest that leaders were overwhelmingly supportive of women in their community using contraception and PAC following spontaneous and induced abortions but expressed negative views towards induced abortion. Given that community leaders can have a strong influence over community beliefs and abortion stigma is thought to discourage women from seeking PAC, these negative attitudes are cause for concern. As such, future program activities should prioritize addressing community leaders’ negative opinions regarding induced abortion in order to increase access to life-saving PAC in this context.

## Introduction

Approximately 222 million women globally have unmet need for contraception, resulting in unintended pregnancies, unsafe abortions, and elevated maternal mortality and morbidity [1]. Such is the case in the Democratic Republic of the Congo (DRC), where modern contraceptive prevalence is just 8%, and the unmet need for contraception is 28% among married women aged 15–49 years [2]. Abortion rates in DRC may be higher than the estimated global average of 35 abortions per 1,000 women aged 15–44 with a rate of 56 per 1,000 women aged 15–49 in Kinshasa; given the restrictive legal^1^ and socio-normative environment in the country, it is probable that a significant portion of these abortions are unsafe and thus pose considerable adverse health risks [3–5]. As post-abortion care (PAC) utilization is low in DRC, complications from unsafe abortions likely contribute to the country’s elevated maternal mortality ratio of 846 maternal deaths per 100,00 live births [2, 3].

While nearly two decades of chronic, intermittent conflict in the eastern regions of DRC have destroyed much of the health infrastructure, exacerbated existing weaknesses in the health system, and likely contributed to low utilization of sexual and reproductive health (SRH) services, socio-normative barriers also impede women’s ability to obtain SRH care [1, 6]. In DRC, socio-cultural norms that favour large families and privilege husbands’ authority to make health and financial decisions for their households can be an obstacle to women’s access to SRH services [7]. A study in rural DRC indicated that both husbands and community members played a significant role in reinforcing unequal gender norms and influencing women’s individual SRH choices; results indicated that conservative attitudes expressed by community members and partners dissuaded women from utilizing contraception, notwithstanding that many participants indicated that they were open to considering or interested in starting a method [8].

Often perceived as the stewards of local traditions, community leaders can wield significant influence over socio-cultural norms and behaviours [9]. As such, gaining and leveraging the support of community leaders can facilitate the success of SRH interventions, especially when the promoted health behaviours deviate from traditional norms [10]. While inadequate engagement of local leaders in health programs has resulted in resistance and programmatic failures [11–13], some health interventions have successfully partnered with local leaders to promote male involvement in maternal healthcare in Kenya and Ghana [10, 14], encourage maternal health service utilization in Malawi [15], and advocate for water and sanitation-related behaviour change in Mali and Zambia [16]. Given their power to influence local customs, community leaders can play a critical role as agents of change in addressing inequitable gender norms and conservative ideologies related to SRH [10, 15, 17].

As socio-cultural and gender norms present barriers to women seeking SRH services in DRC [8], engaging with community leaders could be a successful strategy to expand access to healthcare in these communities. In North and South Kivu, DRC, CARE, the International Rescue Committee (IRC) and Save the Children, in collaboration with the Reproductive Health Access, Information and Services in Emergencies (RAISE) Initiative at Columbia University, support the DRC Ministry of Health (MOH) to improve SRH services. In addition to providing technical assistance to the MOH such as competency-based training, supportive supervision of clinicians, and providing health commodities to improve the quality of care, these partners have dedicated considerable resources to community mobilization in the program areas.

In order to facilitate an enabling environment, project staff engaged with community leaders, defined as individuals who led a civil society group or had the capacity to influence community members’ opinions such as village chiefs, youth leaders and religious leaders. Partners envisioned community leaders taking on a variety of roles to advance SRH access, including linking community members to health services, publicising SRH information, addressing socio-cultural barriers to contraceptive use, actively voicing their support for SRH services during related health campaigns, community events, and town halls, and supporting the implementation of broader health education activities within their communities.

As the majority were male, community leaders were instrumental in engaging men in meaningful discussions concerning gender, social norms, and SRH; leaders further extended the reach of community education by disseminating key SRH messages in traditionally male social spaces, such as bars, radio halls, and sporting events. Some community leaders further supported program activities by participating in partner-facilitated situational analysis that assessed specific cultural, social, and gender normative barriers that limit access to SRH services.

In response to some community leaders’ initial hesitation regarding the importance or appropriateness of expanding SRH services, program staff incorporated values clarification and attitude transformation exercises [18], discussions about the importance of contraceptive and PAC access and review of program data into stakeholder meetings with the community leaders. Through this engagement, partners sought to empower leaders to become SRH advocates within their communities.

RAISE and its partners conducted a program evaluation in 2016–2017 to better understand the barriers and facilitators of access to and use of PAC services. Given the significant role community leaders often play in influencing a community’s socio-cultural norms and acceptance of health services, this paper explores leaders’ perceptions of their role in addressing the issue of unintended pregnancy in their communities.

## Methodology

### Study design, participants and data collection

This manuscript presents findings from key informant interviews (KIIs) undertaken as part of a mixed-methods study conducted in six rural health zones of North and South Kivu. Twelve KIIs were conducted with community leaders, including seven village, association, or neighbourhood chiefs, three principals of secondary schools, and two youth leaders. Two community leaders were purposively selected by program staff in each of the six health zones. Program staff prioritized recruiting both ‘political’ (chiefs) and ‘non-political’ (youth or school) community leaders for the study who had been previously engaged in project activities and who were perceived to have the potential to influence SRH attitudes in key populations, such as among youth and men. Religious leaders were excluded as the program staff was more familiar with their perspectives. All KII participants were male, and further demographic data were not collected. The KIIs took place in September 2016 and April 2017 in the four health zones in North Kivu, and in February 2017 in the two health zones in South Kivu. Following a five-day training, five male interviewers, four in North Kivu and one in South Kivu, used semi-structured interview guides to conduct 25 to 60-min interviews in local languages in private locations. All interviews were recorded with the participant’s consent, transcribed and subsequently translated into French for analysis.

### Data analysis

Electronic files containing the French transcripts were uploaded to NVivo (QSR International Pty Ltd) for coding. Three researchers read the twelve transcripts to identify overarching topics and create a draft codebook organized by general themes and sub-themes. Several transcripts were coded separately by the three researchers using the draft codebooks; after discussing the preliminary results, the researchers revised the codebooks by adding, deleting or collapsing codes as necessary.

Once the codebook was finalized, one researcher coded all twelve transcripts, while the other two researchers each independently coded six transcripts; as such, each transcript was coded by a minimum of two researchers. The primary coder calculated inter-coder reliability, and coding inconsistencies between the researchers were discussed and resolved until the inter-coder agreement was in the 90th percentile range. Finally, the data were interpreted and presented using the respondents’ own words as illustrations.

### Ethical considerations

Verbal informed consent was obtained from all participants. Participant names were not recorded in the transcripts, and only research staff had access to the recordings. The Institutional Review Board of Columbia University and the Institutional Ethical Commission of the Catholic University of Bukavu (DRC) provided ethical approval for this study.

## Results

Results were organized according to several broad themes that emerged during the transcript analysis. These include community leaders’ perceptions of contraception, their perceptions of induced abortion, and their perceived role in addressing unintended pregnancy and induced abortion in their communities.

### Theme 1: community leaders’ perceptions of contraception

In the vast majority of KIIs, participants expressed overwhelmingly supportive views of contraception and birth spacing.

*We are in agreement to do family planning. (Chief, KII 10)*

Very few leaders expressed negative views towards contraception; of the small minority who voiced hesitation, the most common concerns involved providing contraceptives to adolescents or a preference for traditional over modern contraceptives.

*I would like to see that we try to get help from donors who bring us different methods that help us to prevent pregnancies, that they do not bring us modern methods, that we try especially with natural methods. (Youth leader, KII* 8)

Many leaders indicated that they, along with other members of their community, learned about contraception through mobilization and education activities organized by CARE, IRC, or Save the Children.

*Good, in fact, we say thank you to the NGOs that have this component, to know at least how to sensitize people about certain facts like family planning, and especially [NGO] who showed us how we can improve women’s health; even more to show the importance of family planning. (Chief, KII 5)*

Leaders identified a variety of reasons that contributed to their belief that contraception was beneficial for their communities. For example, some leaders indicated that using contraception helped to improve women’s health.

*Ok especially here in [Village] many women’s health is not good, because not everyone has agreed to go to the health center to take a family planning method. ... if you follow the women’s health, you see that the woman who gives birth to eight, ten children, her health will not be good at all … and so not using family planning means the health of the many women here in [Village] is not good.* (Youth leader, KII 12)

Other leaders believed that using contraception to space pregnancies would minimize the risk of miscarriages.

*Either she must stop giving birth immediately to avoid these difficulties, or, she plans her births because often the nurses say that frequently giving birth tires the uterus and that's how miscarriages start to happen. (Chief, KII 7)*

Some leaders indicated that a failure to utilize contraception could result in a litany of health problems for a woman’s current and future children.

*People are afraid that the child's body may not develop properly because the woman will have two children who are almost the same age … . In this case, the first will be malnourished because of this close spacing. (Chief, KII 3)*

Many leaders perceived a clear link between not using contraception and poverty. Some leaders spoke about the difficulty of supporting large families, especially paying school fees, given the lack of employment opportunities and available financial resources in their communities.

*Our community is poor and being poor, we don’t know how to educate so many children, we don’t know how to provide for many children, therefore we find that with this contraceptive method we can manage to plan births. (School principal, KII 4)*

Underpinning these perceived benefits of using contraception was a strong understanding of the relationship between unmet need for contraception and unintended pregnancy. Leaders discussed at length the importance of using contraception to avoid unintended pregnancies and the associated negative consequences.

*When they realize that unwanted pregnancies are occurring, they say no, let's go see the doctor for family planning. (School principal, KII 4)*

### Theme 2: community leaders’ perceptions of induced abortion

The vast majority of leaders readily acknowledged that miscarriage and unintended pregnancies were a regrettable but common occurrence within their communities. However, some leaders initially denied that women in their communities induced abortions.

*Continue the pregnancy because you keep this pregnancy for the State, but to say that you are going to terminate the pregnancy is not good, and currently this habit no longer exists here in our community. (Chief, KII 11)*

While many KII participants were initially reticent, almost all of the leaders ultimately admitted that induced abortion does happen in their communities after probing by the interviewer. Community leaders’ reactions towards induced abortion were overwhelmingly negative. Many leaders perceived abortion to be immoral, unchristian, and violating the norms of their community.

*So that's it, I don’t know how one can describe it, she is rejected completely and we no longer refer to her, she is completely forgotten in society ... she is rejected completely because it's not in our morals … it’s not in our customs. (School principal, KII 2)*

Many community leaders cited the perceived illegality of induced abortion as further justification for their negative attitudes towards the procedure and indicated that they believed women who induced would face criminal repercussions.

*The State has to put her in jail. And there are certain articles of the law that must condemn her, fines she has to pay and the number of days she has to go to prison. (Youth leader, KII 8)*

As a result of the negative views held towards induced abortion, leaders expressed similarly negative perceptions of women who have induced abortions, referencing pejoratives such as witch, prostitute, and murderer. Given these stereotypes, the KII participants believed that women who induced abortions were highly stigmatized and lost value within the community.

*A woman who terminates her pregnancy … she is considered a criminal, she is discriminated against, she is considered as the one who does not have friends because she is a murderer. (Chief, KII 10)*

*We criticize her harshly, we talk a lot about her, she loses her quality and value in the community, and in the neighborhood we take her for someone who practices debauchery, a prostitute. (Chief, KII 3)*

Most leaders predicted that this stigmatization would result in considerable tension between the woman and the community, ultimately leading to marital and familial conflict, discrimination, and societal exclusion.

*I can say that she is no longer useful in the community. It is as if the one who induced abortion is no longer considered in this community. She can move elsewhere since she is a harmful element in the community. (Youth leader, KII 8)*

The overwhelming majority of community leaders believed that young, unmarried women were responsible for a disproportionate share of the induced abortions occurring in their communities. Many leaders expressed that having sex before marriage was stigmatized in their communities and giving birth out of wedlock could damage a girl’s economic, scholastic, and social stability. As such, many KII participants predicted that an unmarried girl would induce an abortion to avoid these social costs.

*Well, it's because these [young] ones have not yet reached the right time to have a child, she has not yet found a husband. Now she will be ashamed of being called a girl-mother. In this case here, she loses her chance to get married or to have a home in the future. (Chief, KII 3)*

A few community leaders went as far as to claim only young, unmarried women would induce abortion, insisting that a married woman would never seek out an abortion.

*It's only girls, it's the girls who find themselves in their little games with their boyfriends, they find themselves pregnant; they have a hard time accepting that because the family will not accept it, it’s a problem for the family, and so they abort in secret. (School principal, KII 2)*

However, upon further discussion the plurality of leaders articulated that factors other than being unmarried could also motivate a woman to induce an abortion, the majority of which related to unintended pregnancy. For example, many community leaders indicated that a married or unmarried woman who became pregnant as a result of rape may induce an abortion. While still viewed negatively, a minority of leaders conceded an understanding of a woman inducing abortion in this situation.

*For example, when she is raped, she gets pregnant when she did not want it when she was planning her life and that leads her to end this pregnancy (Chief, KII 10)*

Other leaders suggested that a married woman, especially a woman who already had many children, was of an advanced age, or had recently given birth, could induce an abortion to avoid the anticipated stigmatization within the community of failing to use contraception to space or limit her pregnancies.

*If it’s an old woman, she will be ashamed, and even the other older women will mock her and say “even at this age you are continuing to have children?” So, she will also induce an abortion at that age. (Chief, KII 6)*

Some leaders highlighted that the widespread poverty present in their communities could motivate a woman to induce an abortion if she believed she would be unable to provide for the child; this theme was often expressed in tandem with concerns regarding unsupportive husbands. Community leaders believed that a woman with an abusive, alcoholic, or unemployed partner may face heightened financial limitations, which could further cause her to seek an abortion.

*In contrast for women, they see the father of the home is a big drinker, he does not have a job, the woman is at the same time mom and dad, herself responsible, for this reason, she decides to have an abortion. (Chief, KII 6)*

While community leaders could identify a variety of situations that might motivate a woman to induce abortion, they were hesitant to explicitly state that this would be an appropriate solution in any situation. Largely, the vast majority of community leaders suggested that regardless of the circumstances, she should carry the pregnancy to term.

*Here, no, she must consent, she must accept, no matter how the husband is, no matter what situation she encounters, then in any case to terminate the pregnancy for a married woman, these are cases that we do not usually hear of. (School principal, KII 2)*

Many leaders referenced the importance of contraception, and suggested that rather than inducing an abortion, women should use a contraceptive method to avoid an unintended pregnancy in the future.

*Instead of interrupting the pregnancy ... you can go and take a contraceptive method at the health facility. (Youth leader, KII 12)*

Even with this hesitancy towards condoning induced abortion, leaders expressed nuanced opinions towards certain cases. For example, a few community leaders indicated that health workers could induce an abortion if the pregnancy endangered the life of the woman.

*It is normal that you terminate a pregnancy so that you do not kill the woman too. This is allowed especially for health workers but not in a house. (Youth leader, KII 8)*

*We see that the woman does not have the strength to give birth without a caesarean section. If she cannot reach this point, we see that it is better to terminate the pregnancy as soon as possible. That can be in the medical field, in their ethics. (Chief, KII 7)*

Additionally, many community leaders supported women’s access to emergency contraception in the case of rape. These individuals clearly recognized that emergency contraception prevented pregnancy rather than terminating an existing pregnancy; indeed, some leaders expressed critical attitudes towards induced abortion in the case of rape while concurrently promoting emergency contraception to avoid pregnancy.

*There are several things to do if she has been raped ... For this reason, we must find out about these victims, teach them to go to a health facility before three days so she receives the medicine to eliminate these diseases that she may have had or so that she does not become pregnant. (Chief, KII 11)*

### Theme 3: community leaders’ perceived roles in preventing unintended pregnancy and responding to induced abortion

With regards to addressing unintended pregnancy and induced abortion in their communities, leaders identified a number of responsibilities they perceived to be inherent to their leadership role. Broadly speaking, these responsibilities included playing a punitive role, promoting conflict resolution, facilitating contraceptive education, and connecting women with PAC services.

#### Punitive role

Although not a majority, some community leaders believed that their role involved ensuring that a woman who induced an abortion faced legal consequences for this perceived illegal action.

*So, when we take this person, we are obliged to put her in the hands of the State to follow up the case. (Youth leader, KII 8)*

Of those KII participants who believed they had a responsibility to support criminal investigations, a considerable portion of their conversation centred upon apprehending those who helped the woman to induce, rather than the woman herself. Community leaders stressed that while a woman who induced an abortion had broken the law, the individuals who had assisted with the procedure were also culpable.

*We do our best to know where she has aborted this pregnancy; if for example it is a nurse who has done it, the prison is close to us and we are in collaboration with the officials, we communicate with them to punish such cases. (Chief, KII 1)*

#### Conflict resolution role

Notwithstanding the negative opinions community leaders professed to hold towards induced abortion, the plurality of leaders believed they had a responsibility to provide emotional support and counselling for people facing interpersonal conflict in their communities, including women who had induced abortions.

*We as leaders … what we do is advise them and if she did the abortion there was a reason either immediate or distant, what we do, we take these two people, we give them advice. (School principal, KII 9)*

Central to the idea of counselling was a belief held by many leaders that a woman who induced an abortion could still be a productive member of society and should not be banished. Multiple leaders stressed that regardless of her actions, a woman who induced an abortion was still a member of their community, thus contrasting beliefs professed earlier regarding the need to ostracize such women. As such, many study participants believed it was their responsibility to provide advice to the woman who had induced, mitigate interpersonal conflict, and ensure that community members could continue to live together in harmony.

*I cannot drive her away, I have to really welcome her and help her because it's not the end of the world, she can do better tomorrow, she can still recover tomorrow. (School principal, KII 2)*

*The first thing I have to do is approach this girl and speak with her. In fact, we cannot ask her to interrupt a pregnancy, that's a good thing. But we cannot send her away from the population. On the contrary, I will talk with her and show her that even though it happened, but she too is a human being, I try to approach her and I approach these others and when I talk with her and I bring her to others, we can also plan a home visit with the neighbors who are having trouble with her. (Youth leader, KII 12)*

Many leaders emphasized that it was vital to approach a woman who had induced an abortion in the spirit of reconciliation in order to avoid adverse outcomes.

*The person can commit suicide because she thinks she is stigmatized, discriminated in this area … , she can be forgiven, we forgive her, we get close to her so she cannot commit suicide. (School principal, KII 9)*

#### Contraceptive counselling role

The vast majority of leaders perceived a strong link between unintended pregnancies and induced abortions within their communities. Some attributed the incidence of induced abortion in their communities to women who had not yet received adequate contraceptive counseling.

*But if this happens to a woman it means that she has not received the teachings … . There are sensitizations that show that the woman can no longer conceive at an undesirable time. (Chief, KII 3)*

As such, many leaders indicated that in order to reduce the number of unintended pregnancies and incidence of induced abortion, women must know how to plan their pregnancies. Sharing information about the benefits and availability of contraception was seen as a priority by many leaders.

*Well, in this village where I am the leader, I am discovering that giving birth to many children, it is necessary that the people in the community access the information from sensitizations and training or education seminar in order to show them how they can limit births. (Chief, KII 3)*

Many KII participants indicated that it was their personal responsibility as a community leader to help expand access to contraceptive services through holding education sessions.

*Our role is what, it is the sensitization of girls ... so we talk to them about family planning starting with all the methods that exist for birth spacing ... for us, our role is raising awareness. (School principal, KII 4)*

In addition to personally counseling women on the importance of family planning, other leaders explained that they would also refer women to health centers to obtain a contraceptive method.

*Yes, to help them, they need to know these methods if they do not want to give birth or if they do not want to have other abortions, they should go to the nurses who know about the methods. We advise them to go to the nurses so that they can explain to them the family planning methods. (Chief, KII 7)*

#### Referral to PAC services role

The vast majority of community leaders believed that women who induced abortions in their communities faced significant health risks as a result of the procedure, including infertility, morbidity, and death. As a result, many leaders emphasized that a woman who had induced must be referred to medical care to prevent negative health outcomes, regardless of their personal views towards abortion.

*What I can do, first of all for a girl who has interrupted a pregnancy, I must first refer her to a health facility, that she goes first to the health center, that she first receives care. After the care you have, there are health facilities that have people who are in charge of psychosocial care, they will help her more in terms of giving advice and counseling, but the first thing I have to do is refer her to the health facility to receive care. (Youth leader, KII 12)*

Some community leaders credited their knowledge of the importance of PAC following a spontaneous or induced abortion and the availability of services to community mobilization efforts within their villages.

*You have to orientate her because she already has advice about abortion, but when others find that they risk dying at home, then when the CHW comes by or the people from the associations find that there is a person at home who has interrupted a pregnancy, they intervene and encourage her to go to the health center for care. (Chief, KII 6)*

Many community leaders believed it was their personal responsibility to ensure that women who induced an abortion received PAC.

*In the first place, I send her to a health facility so that she benefits from care so that she does not have other complications if she is not well cared for. (Chief, KII 1)*

Some community leaders emphasized that all women were entitled to non-discriminatory care, regardless of whether they had experienced a spontaneous or induced abortion.

*We must help for both cases. Because nurses often say that you have to save her life first, something else comes after saving her life. So, the help should not be partial. Take care of everyone please. (Chief, KII 6)*

## Discussion

As the perceived stewards of a community’s traditions, community leaders can have immense influence over how socio-cultural norms are interpreted and enacted within a society [9]. Given the respect accorded to leaders as a result of their positions of political and social power, community leaders may also be influential in spurring health-related behavior change and beliefs among community members, especially regarding traditionally taboo topics [10, 15, 16]. Accordingly, community leaders’ opinions regarding contraception, PAC, and induced abortion can have a significant impact on women’s access to SRH services in North and South Kivu, DRC. Diverging with traditional socio-cultural norms favoring large families in rural DRC [8], community leaders in this study expressed overall positive impressions of contraception, citing numerous associated benefits including improved maternal and child health, reduced poverty, being able to afford children’s school fees, and reducing the incidence of unintended pregnancy and induced abortion.

As opposed to their strong support for contraceptive use, leaders expressed negative opinions regarding induced abortion; as seen elsewhere in the literature, they believed induced abortion was immoral, illegal, and against the socio-cultural norms of their community [19, 20]. While study participants recognized a number of situations that could motivate a woman to induce an abortion, most leaders did not identify scenarios in which they believed inducing an abortion was acceptable. In line with other studies in similar contexts, leaders further predicted that women who induced abortions would be stigmatized in their communities, and would likely encounter criminal and negative social consequences as a result [21, 22]. Given that available evidence suggests that fear of social costs may motivate women to avoid or delay seeking PAC, living in an environment in which induced abortion is highly stigmatized could have adverse health impacts for women in this context [20, 23–25].

In line with the goals of CARE, IRC and Save the Children’s community leader engagement strategies, study participants identified a number of responsibilities associated with expanding access to SRH services that they believed were inherent to their role as community leaders. For example, many leaders placed a heavy emphasis on personally ensuring that women had access to contraceptive counseling and contraceptive methods in their communities. Leaders reported using their respective platforms to publicize the benefits of using contraception and to refer women to health facilities to initiate a method. Notwithstanding their negative personal views towards induced abortion, leaders also championed PAC services, and believed it was their responsibility to encourage women to seek medical care after inducing abortion. Parallel inconsistencies between negative initial reactions towards induced abortion and strong support for life-saving medical care for women who have induced have been observed in other contexts [26]. Similar successes in empowering community leaders to connect women with SRH services have also been observed in other contexts, notably in Malawi with regards to maternal healthcare [15, 27].

In addition to providing information regarding contraception and referring women to health facilities to receive contraceptive counseling and PAC, leaders believed they had a responsibility to mediate interpersonal conflict arising between a woman who had induced abortion and members of the community. Emphasizing that such a woman could still be a productive member of society, community leaders described strategies for diffusing tension, facilitating difficult conversations, and reintegrating women into their community. While their professed commitment to mediating conflict is promising, study participants’ explicit disapproval of induced abortion, support of enforcing punitive sanctions, and promotion of negative stereotypes for women who induced could contribute to low PAC utilization by reinforcing abortion stigma. As observed in other contexts, community leaders can undermine the success of health interventions by using their platform to reinforce conservative socio-cultural norms, such as abortion stigma, even while concurrently ostensibly promoting expanded service delivery [11].

Further engagement with community leaders could be an effective strategy to address conservative beliefs that currently reinforce abortion stigma and presumably contribute to low care-seeking behavior; specifically, implementers should prioritize the integration of values clarification exercises that explicitly challenge community leaders to review their attitudes towards women who induced abortions. When integrated into broader advocacy and programing activities, this approach appears to have been successful in shifting perceptions about induced abortion in other contexts [28–30]. Given leaders’ sway over socio-cultural and gender norms within their communities, empowering leaders to be champions of dismantling abortion stigma could remove normative barriers impeding women’s access to life-saving PAC services [9, 15, 19, 21, 23–25, 31–34].

## Limitations

Although interviewers in this study were not staff of the partner organizations, it is possible that social desirability bias may have influenced community leaders to modify their responses to please the researchers. As the partners have spent years supporting community mobilization and SRH service provision in North and South Kivu, the knowledge, attitudes, and beliefs expressed by the community leaders may not be representative of the general population in DRC. Given that the relatively small sample size, not all cadres of community leaders are represented in the KIIs for each of the six health zones. All respondents were male; female leaders may have different opinions.

## Conclusion

Although collaborating with community leaders is widely accepted as a best practice in health programming, limited rigorous research has been undertaken regarding their role in improving SRH access. Results from this study suggest that when thoughtfully engaged, community leaders can be empowered to become advocates for SRH. While community leaders in North and South Kivu, DRC championed use of contraception and PAC services, the vast majority espoused negative opinions regarding induced abortion. Given the hypothesized link between the presence of abortion stigma and care-avoidance behavior, coupled with the influence community leaders often command over the socio-cultural and gender norms of a society, further engagement and values clarification exercises with leaders must be prioritized in order to increase PAC utilization.

## Data Availability

The study materials and instruments used during the study are available from the corresponding author on reasonable request.

## Abbreviations

DRC: Democratic Republic of the Congo
IRC: International Rescue Committee
KII: Key informant interview
MOH: Ministry of Health
NGO: Non-governmental organization
PAC: Post-abortion care
RAISE: Reproductive Health Access, Information and Services in Emergencies Initiative
SRH: Sexual and reproductive health

## Footnotes

1 DRC has signed and ratified the Protocol to the African Charter on Human and People’s Rights on the Rights of Women in Africa (Maputo Protocol), a legally binding treaty which authorizes abortion in cases of rape, incest, foetal impairment and to preserve the mental or physical health or life of the woman. However, at the time this research was conducted, necessary changes to national law had not yet been made and the law was generally interpreted to permit abortion only to save the life of a woman.
